# Effects of a nurse-led medication self-management programme in cancer patients: protocol for a mixed-method randomised controlled trial

**DOI:** 10.1186/s12912-016-0130-1

**Published:** 2016-02-08

**Authors:** Hiroko Komatsu, Kaori Yagasaki, Takuhiro Yamaguchi

**Affiliations:** Faculty of Nursing and Medical Care, Keio University, 35 Shinanomachi, Shinjuku-ku, Tokyo 160-8582 Japan; Division of Biostatistics, Tohoku University Graduate School of Medicine, 1-1 Seiryo-machi, Aoba-ku, Sendai, Miyagi 980-8574 Japan

**Keywords:** Metastatic breast cancer, Adherence, Patient-centred care, Shared decision making, Concordance, Patient preference, Self-management

## Abstract

**Background:**

With the widespread use of orally administered anticancer agents, self-management by cancer patients is inevitable, and adherence to medication is becoming the centre of interest in oncology.

**Methods/Design:**

This mixed-method study is a two-phased approach with a combined quantitative and qualitative design. In the first phase, we will conduct a prospective randomised controlled study to assess the effects of a nurse-led medication self-management programme for patients receiving oral anticancer treatment. Patients with metastatic breast cancer, who have been newly prescribed an oral chemotherapy or a targeted therapy agent will be enrolled in the study. The participants will be randomly assigned to either the medication self-management support programme group (intervention group) or the conventional care group (control group). This will be an open-label study; therefore, neither the patients nor the nurses will be blinded. Nurses will provide patients in the intervention group with information by using the teach-back method, help patients set a goal based on their preferences, and solve problems through follow-up counselling. The primary outcome measure is adherence to medication, to be measured on the basis of the medication possession ratio (MPR), which is the ratio of the number of days of medication supply to the total days at a specified time interval. We hypothesize that the intervention group will have an MPR of ≥90 % that is significantly higher than that of the control group. Secondary outcome measures include self-efficacy, quality of life, psychological distress, severity and interference of symptoms, patient satisfaction, emergency department visits, and hospital admissions. In the second phase, we will conduct focus-group interviews with intervention nurses, and perform a content analysis to understand their role and challenges these nurses will face in the programme while improving patients’ medication adherence.

**Discussion:**

The present study will be the first Japanese study to evaluate the effects of medication self-management support provided by nurses to patients with metastatic breast cancer who are receiving oral anticancer treatment. The study is characterised by a unique patient-centred approach aiming to help patients manage their medication based on their needs and preferences, with both quantitative and qualitative evaluations. The findings will contribute to the facilitation of medication management in cancer patients.

**Trial registration:**

UMIN Clinical Trials Registry (UMIN-CTR), Japan, UMIN000016597. (27 February 2015).

## Background

Metastatic breast cancer (MBC), also referred to as secondary or advanced breast cancer, is defined as breast cancer that has spread to other parts of the body [1]. MBC is currently incurable; however, it can be managed as a chronic disease with appropriate treatment strategies [2]. The systematic treatment options for MBC are endocrine therapy, chemotherapy, and biological (targeted) therapy [3]. The goals of care are to control disease progression, extend survival, optimize symptom management, and enhance the quality of life [3–5]. A previous study suggested that patients with MBC are willing to accept substantial risks of side effects in exchange for potential survival benefits [5].

As oral anticancer agents are becoming more common, a critical shift has occurred from clinic-based healthcare provider-administered management to home-based self-administered management, with patient adherence becoming increasingly important [6]. There are very few studies on adherence to medication in patients with MBC, and the results are inconsistent. Although Figueiredo et al. reported high adherence to capecitabine in patients with MBC [7], Di Bonaventura et al. addressed nonadherent beheviours due to forgetfulness and intolerance of side effects in an internet-based study on multiple anti-cancer drugs [5]. Schulman-Green et al. [8] addressed barriers to self-management in patients with MBC, including symptom distress, difficulty in obtaining information and lack of knowledge about the course of cancer. Cancer survivors have unmet needs concerning personal control (e.g., maintaining autonomy and independence), physical problems (e.g., pain and symptoms), and education/information (e.g., lack of knowledge); breast cancer patients identified more unmet needs than other survivors [9]. Furthermore, anxiety and symptoms of depression increase with the increasing incidence of cancer recurrence [10]. When concerns outweigh necessity beliefs, nonadherence occurs according to the balance theory of the necessity-concerns framework [11, 12]. Because many problems specific to MBC are left unanswered, patients with this disease frequently feel a sense of abandonment and isolation, including feelings of uncertainty [13], a lack of control, and poor emotional functioning [14]. With this background, patients with MBC are at risk of nonadherence to medication.

A systematic review on adherence-enhancing interventions for oral chemotherapy suggested that educational and consultation interventions are promising [15]. The models of concordance and shared decision making have emerged as patient-centred approaches [16]. In concordance, a therapeutic alliance is established between the healthcare professional and the patient through encouraging patients to discuss concerns about medications and preference for treatment and participation in decision making. Similarly, the patient and the healthcare professional share knowledge and experience on the available options to make a decision jointly in shared decision-making. As the needs of MBC patients vary greatly, care and support should be tailored to each individual patient, and the patients are encouraged to participate in the decision-making process [4, 17]. It is critical for healthcare professionals to listen to and understand the patients’ concerns, beliefs, preferences, and expectations, and to confirm their understanding and commitment to the treatment [18]. In terms of the patients’ control over medication management, healthcare professionals should focus on helping the patients uncover important issues, set a goal, and solve any problems [19].

The importance of an individualised approach specific to MBC and respecting the patient’s preferences is emphasised in the international consensus guidelines for advanced breast cancer [3]. Although the subjects of the study were not MBC patients, positive results were reported concerning the effect of patient preference-based interventions on adherence [20] and outcomes [21]. A systematic review of quantitative and qualitative studies revealed that providing written information only is not useful; patients do not value written information about medication and do not want this type of information as a substitute for discussion [22]. Teach-back has been used as an educational strategy for patients with chronic disease. This involves asking patients to repeat the key points of a topic or instruction to ensure their understanding of the information provided by the healthcare professionals [23]. This process helps motivate patients to adhere to medication and self-management [24].

With the increase in oral therapies, nurses need to spend more time focusing on patients’ adherence to medication [25] by providing proactive care [26]. Nurses play an important role in educating patients about their treatment and the management of side effects [27], monitoring adherence by identifying potential barriers and implementing intervention strategies [28], and helping patients recognise when to seek professional help [25]. Compared with adherence to hormone therapy in cancer patients [29–33], adherence to oral chemotherapy and targeted therapy is a relatively new area of research. Two studies have reported the significance of nurse intervention, over the telephone in improving adherence to oral chemotherapy; however, one was a feasibility study and the other was a randomised study with a small sample size [34, 35]. Spoelstra et al. [36] suggested the importance of patient education provided by nurses for promoting adherence and managing symptoms. The effect of an intensified pharmaceutical care, including patient education and consultation, has also been reported for the improvement of adherence to capecitabine chemotherapy in patients with breast or colorectal cancer [37].

Few studies have reported on interventions to improve adherence to oral chemotherapy; moreover, studies about oral chemotherapy specific to patients with MBC are rare. The current study focuses on adherence to oral chemotherapy and targeted therapy in an MBC population. We will highlight teach-back, goal setting based on patient preferences, and problem solving through follow-up counselling by nurses under the concept of concordance and shared decision making. We will also conduct a prospective randomised controlled trial on patient-centred intervention to facilitate medication management in MBC patients at three cancer centres in Japan. We will also perform qualitative evaluations on the programme based on the perceptions of intervention nurses.

### Objectives

The objectives of this study are to determine the effects of a patient-centred medication self-management support programme in patients with MBC undergoing oral anticancer treatment, and to evaluate the programme’s effectiveness based on the perceptions of intervention nurses. We hypothesize that the intervention group will have a medication possession ratio (MPR) ≥90 % that is significantly higher than that of the control group.

## Methods/Design

### Study design

**Fig. 1:**
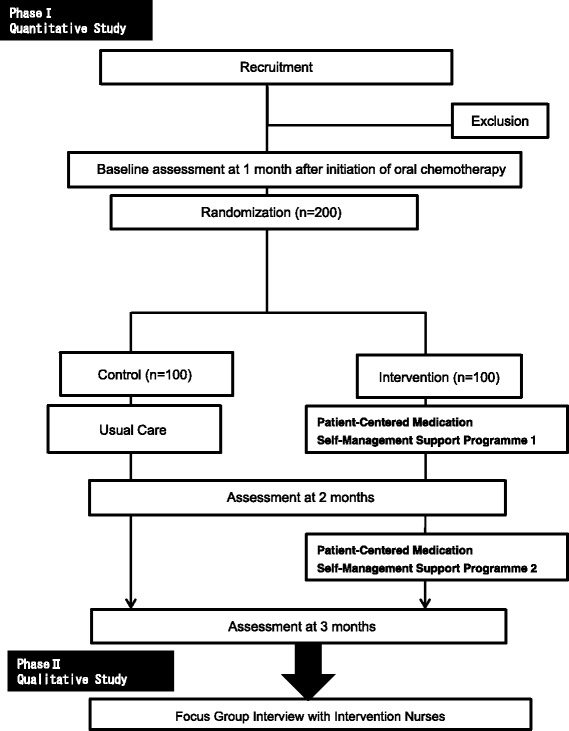
Schema of study design

The study is a two-phased mixed-method approach, with a prospective randomized, parallel-group, two-arm, open-label, three-centre study and a qualitative study using a focus-group interview. A summary of the study design is given in Fig. 1.

### Study setting

The study will be conducted at three cancer centres in the greater Tokyo area in Japan: the National Cancer Centre Hospital (600 beds), the National Cancer Centre Hospital East (425 beds), and the Kanagawa Cancer Centre Hospital (415 beds).

#### Phase I: An intervention study

##### Recruitment

Primary physicians will identify a sample of eligible participants from outpatient lists of three cancer centre, and recruitment will be performed by nurse investigators at the outpatient clinics before the commencement of oral chemotherapy. Participants will be eligible for the study if they (i) are 20–90 years of age, (ii) have MBC, (iii) can complete a questionnaire in Japanese, (iv) have been newly prescribed with oral chemotherapy or target therapy medication, (v) can manage their medication without any assistance, and (vi) have been given a prognosis of ≥3 months, as determined by their primary physician. Patients will be excluded if they (i) have cognitive impairment or mental disorder, (ii) have brain metastasis, (iii) are receiving infusion therapy, or (iv) are participants in another study. The nurse investigators will provide research information to those who are interested in the study, and will obtain written consent with their potential participants’ signatures. Then, the nurse investigators will send enrolment forms to a data centre through e-mail. The participants will be enrolled after a final determination of eligibility at the data centre. The target sample size is 200 (100 per arm), and the number of planned patients for recruitment at the three cancer centres　(the National Cancer Center Hospital, the National Cancer Center Hospital East and the Kanagawa Cancer Center) will be 64, 64, and 72, respectively.

#### Phase 2: A Qualitative study

After the phase 1 intervention study, nurse investigators will recruit 5 to 10 intervention nurses from each facility to participate in the phase 2 qualitative study at a research meeting. Detailed written information and oral explanation of the study were given to those who were interested in the phase 2 study. Written consent will be obtained from the participants.

##### Randomisation and blinding

The participants will be randomized to either the medication self-management programme group (intervention group) or the conventional care group (control group) at a 1:1 ratio. The Joint Centre for Researchers, Associates, and Clinicians (JCRAC), an independent, non-profit organisation with extensive experience in conducting clinical trials, will conduct the randomisation by using a computerised random number generator. Randomisation will be stratified according to age (<40 vs. 40≦), treatment regimen (Xeloda vs. Xeloda + Tykerb vs. TS-1), and cancer centre. A database containing patient names will be encrypted and sent to the JCRAC. This is an open-label study; therefore patients, nurses, and investigators will not be blinded.

### Intervention

#### Conventional care

Both the control group and the intervention group will receive conventional care from healthcare professionals at the outpatient clinics in each of the participating cancer centres. This includes i) explanation on oral chemotherapy and physical evaluation during the course of the treatment provided by the physician, and ii) instructions on the medication including procedure, frequency, dose, and the steps to take if the patient overdoses or forgets to take the medicine; and (iii) information and contact details with regards to treatment-related toxicity and emergencies provided by the pharmacist; a leaflet will be provided on commencement of treatment. Outpatient nurses will provide psychological support to patients only on request, and nurses providing conventional care will not be trained for the intervention programme.

#### Intervention protocol

We developed the patient-centred medication self-management support programme to consist of three elements: information giving by using teach-back, patient preference, and follow-up by a nurse under the concept of concordance and shared decision making [16, 38–40]. The goal of the programme is to improve adherence to medication and self-management. In addition to the conventional care, the intervention group will receive two sessions of the patient-centred medication self-management support programme conducted by trained nurses in the clinic at 1 and 2 months after enrolment. Each session will take 20–30 min. In the first session, patients will be asked for feedback, in their own words, on what they understand about the medication information provided by the healthcare professionals. Through repeating back the key points of the instructions, the nurse will help the patient recognize whether he or she understands the information correctly and provide motivation toward medication self-management. The patient and nurse will share information and needs, and set a goal on the basis of the patient’s preferences, to facilitate medication management. In the second session, the nurses will review the patients’ management of their medication, and help them solve any problems concerning medication management. The follow-up counseling is restricted to scheduled appointments.

### Intervention nurses

Three to five oncology nurses specialized in chemotherapy will deliver the intervention at each participating centre. These intervention nurses will have had more than 5 years of experience in chemotherapy and will have agreed to participate in the study. To standardize intervention, a 120-min training workshop programme will be implemented for nurses who will be responsible for delivery of the intervention prior to the study. The training programme will include (i) self-management of oral administration, and the concepts of concordance and shared decision making as a patient-centred approach; (ii) basic knowledge and optimal management of oral chemotherapy and targeted therapy; and (iii) teach back and effective communication skills (Table 1).

**Table 1:** Training of intervention nurses

Learning contents
1. Understanding the basic theory of medication self-management of oral anticancer agents, and the concept of concordance and shared-decision making as a patient-centred approach.
a. Self-management theory
b. The concept of concordance and shared-decision making as a patient-centred approach
2. Understanding the basic knowledge and optimal management on oral anticancer agents.
a. Treatment regimen and management of side effects
b. Daily management of anticancer agents
c. Steps to be taken if the patient forgets to take a medicine or overdoses
3. Acquire the teach-back and effective communication skills.
a. Communication skills
b. Teach-back skills
c. Confirmation of the patient’s understanding and provision of essential information
d. How to share and review patient preferences in medication management
Q&A (Total 120 minutes)

### Data collection

#### Primary outcome measure

The primary outcome is adherence to medication at 3 months after the commencement of oral chemotherapy or target therapy, as ascertained from the MPR, which is the ratio of the days of medication supply to the total days at a specified time interval, calculated from the medical chart. An MPR of 0 would indicate that no medication had been taken. In this study, an MPR of 90 % will be considered as indicating good adherence [41]. Medication adherence will also be assessed by the Japanese version of the Morisky Medication Adherence Scores (MMAS-8). MMAS-8 is an eight-item self-reporting measure of medication-taking behaviour [42]. The first seven items have a dichotomous response (yes or no); the eighth item has a five-point Likert scale response. The total score of MMAS-8 ranges from 0 to 8. A higher score represents higher adherence to medication.

#### Secondary outcome measures

A number of secondary outcome measures will also be assessed.

##### Self-efficacy

We will use the Japanese version of the General Self-Efficacy (GSE) Scale to assess self-efficacy [43]. The GSE Scale is designed to assess optimistic self-beliefs to cope with a variety of difficult demands in life; it includes10 items, such as ‘I can always manage to solve difficult problems if I try hard enough’ [44]. The response scale ranges from ‘not at all true’ (1 point) to ‘exactly true’ (4 points), and the response scores of all 10 items will be summed. Higher scores indicate higher perceived general self-efficacy.

##### Quality of life

Quality of life will be measured by using the Japanese version of the Functional Assessment of Cancer Therapy-Breast (FACT-B) [45]. This is a 36-item self-reported questionnaire consisting of four subscales: physical (7 items), family/social (7 items), emotional (6 items), functional well-being (7 items), and Breast cancer (9 items) [46, 47]. A 5-point Likert scale ranging from ‘not at all’ (0) to ‘very much’ (4) is used. Higher scores indicate higher quality of life.

##### Psychological distress (K6)

We will use the Japanese version of the Kessler 6 (K6) to assess psychological distress [48]. The K6 is a self-administered tool used for screening mental health issues by asking 6 questions [49]. Individuals will be asked the frequency of feeling nervous, hopeless, restless or fidgety, so depressed that nothing could cheer you up, that everything was an effort, and worthless, during the last 30 days. A score of 10 or more is used to indicate non-specific serious psychological distress.

##### Symptoms severity and interference

We used the Japanese version of the M.D. Anderson Symptom Inventory to assess perceived symptom severity and interference [50]. This is a multi-symptom patient-reported outcome measures for clinical and research use and applies broadly across cancer types and treatments [51]. There are 13 symptom severity items (pain, fatigue, nausea, disturbed sleep, emotional distress, shortness of breath, lack of appetite, drowsiness, dry mouth, sadness, vomiting, difficulty remembering, and numbness or tingling) and severity is rated on a numerical rating scale, from 0 ‘not present’ (0 points) to ‘as bad as you can imagine’ (10 points) [52].There are 6 symptom interference items (general activity, mood, walking ability, normal work, relations with other people, and enjoyment of life), and interference is rated on a numerical rating scale, from ‘did not interfere’ (0 points) to ‘interfered completely’ (10 points). A component score for both symptom severity and interference will be obtained by taking the average of the two scores. Higher scores represent greater symptom severity and interference.

##### Patient satisfaction

Patient satisfaction of the medication self-management support programme is measured by two questions developed for this study. 1) Are you satisfied with the medication self-management support programme? and 2) Do you want to continue to receive support from healthcare professionals? The five response options range from ‘not at all satisfied’ to ‘extremely satisfied’. A higher score indicates greater satisfaction.

Baseline measures will be assessed at 1 month after the initiation of oral chemotherapy before randomisation. Health service visit, including emergency department visits and admissions will be collected from medical records, and only treatment change (discontinuation/dose reduction/oral chemotherapy free interval) will be described in a case report form. The study uptake rates and compliance with the intervention will be recorded. Table 2 summarises the measures and timings for each measurement.

**Table 2:** Measures and timings for measurements

Measure	Questionnaire	Baseline	2 months	3 months
Demographics	Patient Questionnaire	○		
Medication adherence	MPS	○	○	○
	MMAS-8	○	○	○
Self-efficacy	GSE	○	○	○
QOL	FACT-B	○	○	○
Psychological distress	K6	○	○	○
Symptom severity/interference	MD Anderson Symptoms Inventory	○	○	○

##### Phase II: A Qualitative study

We will conduct a focus-group interview (80 – 90 min.) with 15 - 18 intervention nurses. Investigator nurses (HK or KY) will take the role of facilitator, and use a semi-structured interview guide to explore the role and challenges of the nurses in the patient-centred medication self-management support programme. The questions will include the following issues: i) perceived usefulness of the programme for MBC patients receiving oral chemotherapy, ii) perceived challenges in applying the programme to practice, and iii) perceived patients’ responses to the programme. The focus-group discussion will be recorded and transcribed. Qualitative content analysis will be used, which will include definition of the analytical unit contents, analytical steps taken by a category system, re-checking the category system by applying it to theory and material, and interpretation of the results in relation to the main problem and issue [53].

### Data management

Completed questionnaires will be coded with a participant identification (ID). All research-related documents will be linked by using the participant ID. All study data will be sent to the data centre in PDF format, and will be entered into an Excel file by the data managers at the data centre. The original questionnaires will be stored securely at locked cabinets in outpatient clinics or the nursing department.

### Sample size calculation

The programme will be considered as effective if the proportion of patients who maintain ≥90 % MPR is significantly higher in the intervention group than in the control group, at the completion of 3 months of oral chemotherapy. Previous studies have assumed that adherence has been achieved if the MPR is 70 % in the intervention group and 50 % in the control group [32, 41]. Prospective power analysis revealed that 93 patients were needed in each group for 80 % power at an alpha level of 0.05. Therefore, we plan to recruit 100 patients in each group, for a total of 200 patients.

### Statistical methods

The analysis will be done on the basis of intention-to-treat principle. For the primary outcome, the proportion of patients who maintained ≥90 % MPR in each group at 3 months will be estimated and compared, with adjustments made for allocation factors. If a statistically significant difference is found, our intervention will contribute to medication management in MBC patients. For the other end points, summary statistics will be calculated at each measurement time point in each group and compared between groups. The significance level will be set at 0.05.

### Research ethics approval

This study was approved by the internal review board of Faculty of Nursing and Medical Care, Keio University (No. 218), and by the three cancer centres. All participants will receive a verbal and written description of the study and be asked to provide informed consent.

## Discussion

The present study will be the first Japanese study to evaluate the effects of a medication self-management support programme on patients with MBC undergoing chemotherapy or targeted therapy. It is characterized by a unique patient-centred approach with the principles of concordance and shared decision-making. A partnership between the patient and the healthcare professional is important in order to facilitate sharing of knowledge and experience with each other to reach an agreement on treatment [54]. Nurses will help patients understand information by using teach-back, set goals with the patients concerning medication management based on patients’ needs and preferences, and help patients solve problems through follow-up counselling.

This mixed-method approach will allow an in-depth understanding of the effects of the medication self-management support programme. In the first phase, the primary outcome in the quantitative study will be assessed by using both objective (MPR) and subjective (MMAS-8) measures. Enhanced patient adherence to medication due to the intervention may lead to positive effects, which will be measured by using a wide range of outcomes including self-efficacy, psychological distress, symptoms, and patient satisfaction. In the second phase, the study will adopt a qualitative evaluation by using content analysis, which will provide practical information from the perspectives of the intervention nurses. A training programme has been developed, and to minimise variability among interventionists, the intervention nurses will be trained on medication self-management of anticancer agents before the study. This programme will also be useful when the intervention is applied in the clinical setting.

It should be noted that performance bias might occur owing to the open-label design of this study. The nature of the study, however, allows for refinement of the intervention in practice. When a patient is aware that adherence is being evaluated, overestimation of adherence in the self-reporting method is a concern. Although the MPR is commonly used to measure adherence, it is only a representation for actual medication use; we do not know whether the medication is actually taken or not. The present study will be carried out at three cancer centres; therefore, further research is needed in other settings to assess generalizability.

As the number of patients undergoing oral chemotherapy continues to increase, there will be a growing need for evidence-based medication self-management support. The present study has the potential to facilitate medication management not only in patients with MBC, but also in those with other types of cancer. The proposed intervention enhances the role of nurses in supporting patients and can be integrated into clinical practice.

## Abbreviations

FACT-B: functional assessment of cancer therapy-breast cancer
GSE: general self-efficacy
JCRAC: Joint Centre for Researchers, Associates and Clinicians
K6: Kessler 6
MMAS-8: Morisky Medication Adherence Scores -8 items
