# Erratum to: Effects of a nurse-led medication self-management programme in cancer patients: protocol for a mixed-method randomised controlled trial

**DOI:** 10.1186/s12912-016-0180-4

**Published:** 2016-10-24

**Authors:** Hiroko Komatsu, Kaori Yagasaki, Takuhiro Yamaguchi

**Affiliations:** 1 Faculty of Nursing and Medical Care, Keio University, 35 Shinanomachi, Shinjuku-ku, Tokyo, 160-8582 Japan; 2 Division of Biostatistics, Tohoku University Graduate School of Medicine, 1-1 Seiryo-machi, Aoba-ku, Sendai, Miyagi 980-8574 Japan

## Erratum

Upon publication, the below errors were noticed in the original version of the article [1].

1. In the title of the article, ‘randomised’ should be spelt ‘randomized’
  2) i) In Table 2, ‘MMAS-8’ should instead read ‘cMMAS-8’
  ii) In Table 2, the following sentence should be included as a footnote: “Use of the cMMAS is protected by US copyright laws. Permission for use is required. A license agreement is available from: Donald E. Morisky, ScD, ScM, MSPH, Professor, Department of Community Health Sciences, UCLA School of Public Health, 650 Charles E. Young Drive South, Los Angeles, CA 90095-1772.”
  iii) In Table 2, there should be check marks under the ‘Baseline’, ‘2 months’, and ‘3 months’ columns in the PDF as per the HTML
  3) In the Acknowledgements, the following sentence should be included as the second paragraph: “Use of the cMMAS is protected by US copyright laws. Permission for use is required. A license agreement is available from: Donald E. Morisky, ScD, ScM, MSPH, Professor, Department of Community Health Sciences, UCLA School of Public Health, 650 Charles E. Young Drive South, Los Angeles, CA 90095-1772.”
  4) In citation 42, ‘Morisky DE, Green LW, Levine DM. Concurrent and Predictive Validity of a Self-Reported Measure of Medication Adherence and Long-Term Predictive Validity of Blood Pressure Control. Med Care. 196:24:67-74’ should read:
  A. Morisky DE, Ang A, Krousel-Wood M, Ward H. Predictive Validity of a Medication Adherence Measure for Hypertension Control. Journal of Clinical Hypertension 2008; 10(5):348-354.
  B. Krousel-Wood MA, Islam T, Webber LS, Re RS, Morisky DE, Muntner P. New Medication Adherence Scale Versus Pharmacy Fill Rates in Seniors With Hypertension. Am J Manag Care 2009; 15(1):59-66.
  C. Morisky DE, DiMatteo MR. Improving the measurement of self-reported medication nonadherence: Final response. J Clin Epidemio 2011; 64:258-263. PMID: 21144706

**Table 2:** Measures and Timings for Measurements

Measure	Questionnaire	Baseline	2 months	3 months
Demographics	Patient Questionnaire	○		
Medication adherence	MPS	○	○	○
	cMMAS-8	○	○	○
Self-efficacy	GSE	○	○	○
QOL	FACT-B	○	○	○
Psychological distress	K6	○	○	○
Symptom severity/interference	MD Anderson Symptoms Inventory	○	○	○
